# Classifying Severe Acute Kidney Injury in Cirrhosis: Implications for Mortality?

**DOI:** 10.34067/KID.0000000637

**Published:** 2025-02-27

**Authors:** Daniel Pascal Msilanga, Yuenting Diana Kwong

**Affiliations:** 1 Department of Internal Medicine, Nephrology Unit, Muhimbili University of Health and Allied Science, Dar es Salaam, Tanzania; 2 Division of Nephrology, Department of Medicine, University of California, San Francisco, San Francisco, California

**Keywords:** AKI, liver failure

Severe AKI requiring KRT in patients with cirrhosis is associated with high rates of mortality.^1^ AKI in patients with cirrhosis can be classified into common phenotypes on the basis of etiology: prerenal (volume-mediated), intrinsic (ischemic or nephrotoxic), or postrenal (obstructive) causes. Unique to patients with cirrhosis, hepatorenal syndrome (HRS), a specific type of prerenal AKI, may also occur as a result of splanchnic vasodilation, leading to a reduction of effective arterial blood volume, compensatory renal vasoconstriction, and eventually decreased renal blood flow.^2^ Differentiating HRS versus non–HRS-related AKI is challenging, yet has significant therapeutic implications. US and European guidelines recommend intravenous albumin infusion combined with vasoconstrictors, such as terlipressin in HRS-AKI.^3,4^ However, limited studies exist on the prognostic implications of mortality in those with HRS versus non-HRS AKI, especially among patients with cirrhosis who require KRT.

In this issue of Kidney360, using data from 374 patients with AKI that required KRT in 2019 from the HRS-Harmony consortium that collects data from 11 US Hospitals, Cama-Olivares *et al.* evaluated the association of AKI etiology with 90-day mortality.^5^ HRS-AKI was identified as the AKI etiology in 17.4%, while 82.6% were classified as having non-HRS AKI, with 62.6% specifically diagnosed with acute tubular necrosis (ATN). The total 90-day mortality rate was 59.6%, with 49.2% of those with HRS-AKI and 65.8% with ATN dying within the timeframe. A Fine-Gray analysis, which accounts for the competing risk of liver transplantation, showed that HRS versus non-HRS AKI were not significantly associated with increased 90-day mortality (subdistribution hazard ratio, 1.36; 95%, 0.95 to 1.94).

Of the total cohort of 374 patients with cirrhosis who were initiated on KRT, 26.5% were listed for liver transplant and 17.1% were transplanted, with 90-day mortality being significantly lower in those listed versus those who were not (28.3% versus 70.9%). Compared with patients without HRS-AKI, those with HRS-AKI received more vasoconstrictors (81.5% versus 67.9%). Those with HRS-AKI were more frequently listed and received liver transplants. Key parameters associated with 90-day mortality include vasopressor use for shock, initial dialysis modality of continuous (versus intermittent) KRT, high disease severity score, and not being listed for liver transplant.

This study may be one of the larger multicenter contemporary cohort of patients with AKI who require KRT with cirrhosis. Allegretti *et al.* previously analyzed data from 472 patients with cirrhosis who were initiated on KRT at five hospitals between 2005 and 2015 and found a 6-month mortality of 84% in patients with HRS and 85% in patients with ATN.^6^ Similar to Cama-Olivares *et al.*, Allegretti *et al.* found no significant difference in outcomes between those diagnosed with HRS versus ATN regardless of transplant listing status. Both studies assessed AKI etiology using two adjudicators and a third referee providing a tiebreaker diagnosis when necessary. Overall, the data seem to signal that diagnosis of HRS versus non-HRS AKI may not be associated with prognosis; instead, mortality in this population is likely driven by disease severity.^5–7^

AKI adjudication may be difficult in cirrhosis because patients often have overlapping clinical syndromes; ATN and severe forms of HRS-AKI remain difficult to distinguish in clinical practice. Traditionally, HRS-AKI is a diagnosis of exclusion characterized by the absence of where patients have no current or recent use of nephrotoxins exposure, no signs of shock, and no signs of structural kidney injury, as indicated by proteinuria, microhematuria, and/or abnormal renal ultrasound.^3^ In this study, fractional excretion of sodium, collected in 61% of the population, did not significantly differ between groups. Urine microscopy may point to ATN with tubular epithelial casts, but samples in severe AKI are often unavailable during severe oliguria. Growing recognition of the potential benefit that patients with existing structural kidney damage may still derive from HRS treatment has led to an updated definition of HRS-AKI by the Acute Disease Quality Initiative and International Club of Ascites and evidence of pre-existing structural damage no longer precludes the diagnosis of HRS-AKI.^8^

Current evidence indicating the lack of association between AKI etiology and mortality may be limited by the retrospective nature of the existing studies and residual confounding factors. The suspected etiology of AKI likely modified interventions and affected mortality outcomes. Results may be further biased as not all patients with decompensated cirrhosis are initiated on KRT. Generalizability is also restricted because these studies were conducted solely in US hospitals with abundant resources and access to transplant services centers. By expanding from five hospitals to 11 US hospitals, Cama-Olivares *et al.* more comprehensively described mortality rates in patients with cirrhosis and AKI that require KRT while accounting for the heterogeneity of KRT initiation practices in different institutions. The study strongly demonstrates that, even in resource-abundant settings, mortality of patients with cirrhosis with AKI that require KRT is high. In settings with limited access to transplant, the mortality rates are suspected to be even higher.

KRT is often viewed as a supportive therapy for patients with cirrhosis who are candidates for liver transplant, but remains highly controversial for patients who are not. Current evidence supports that patients with HRS requiring KRT have comparable outcomes with those with cirrhosis and non-HRS AKI, such as ATN also requiring KRT. The study results challenge the common assumption that HRS carries a worse prognosis than other AKI etiologies, underscoring the need to focus on overall illness severity. For patients who are not liver transplant candidates, the decision to initiate dialysis in patients with cirrhosis and AKI should not be based on the suspected etiology of AKI. Instead, a comprehensive care team approach involving palliative care expertise is recommended to initiate discussions with patients and their caregivers regarding the goals of therapy in the face of poor long-term prognosis. For patients who undergo KRT and are not liver transplant candidates, it remains unclear whether these patients achieve sufficient longevity to counterbalance the detriment to the quality of life as a result of undergoing dialysis, which may lead to worsening fatigue, cramps, and potential for increased instability during ultrafiltration for volume optimization. Further studies are needed among patients who choose palliative alternatives.

Ultimately, this HRS-Harmony study, which reports data from 2019, presents valuable baseline information on outcomes in patients with cirrhosis and AKI requiring KRT before the US rollout of terlipressin. In 2022, the Food and Drug Administration approved terlipressin for the treatment of HRS-AKI after the results of the A Multi-Center, Randomized, Placebo Controlled, Double-Blind Study to Confirm Efficacy and Safety of Terlipressin in Subjects With Hepatorenal Syndrome Type 1 (CONFIRM) trial, where 300 patients with HRS were assigned in a 2:1 ratio of terlipressin or placebo up to 14 days.^9^ The CONFIRM trial showed no significant differences in 90-day all-cause mortality. As such, the mortality rates between severe HRS-versus non-HRS AKI may not change significantly in the future.

However, CONFIRM showed significantly higher rates of HRS reversal among those with terlipressin (32% versus 17%). Notably, the trial excluded patients who were initiated on dialysis and patients who were found to have tubular epithelial casts. The future remains undetermined as to whether terlipressin will be routinely deployed in those with AKI with suspected overlapping HRS and ATN who are at the cusp of needing KRT. Perhaps terlipressin use in these patients may lead to less ongoing ischemic damage and improve a patient's chance of renal recovery in the setting of cirrhosis, eventually limiting mortality complications related to KRT. However, as terlipressin use becomes more widespread, the benefits of renal recovery must be carefully weighed against the risks of respiratory death. Identifying the subpopulations of those with cirrhosis of AKI who are at low risk of pulmonary compromise with high possibility of renal recovery with terlipressin seems to be the forthcoming challenge. With rigorous scientific guidance in the optimal population for terlipressin use, the signal in decreased mortality may eventually follow.
